# The Multifaceted Functions of Prion Protein (PrP^C^) in Cancer

**DOI:** 10.3390/cancers15204982

**Published:** 2023-10-13

**Authors:** Roland Abi Nahed, Hasan Safwan-Zaiter, Kevin Gemy, Camille Lyko, Mélanie Boudaud, Morgane Desseux, Christel Marquette, Tiphaine Barjat, Nadia Alfaidy, Mohamed Benharouga

**Affiliations:** 1 U1292, Laboratoire de BioSanté, Institut National de la Santé et de la Recherche Médicale (INSERM), F-38058 Grenoble, France; rolandabinahed@gmail.com (R.A.N.); hasan.safwan-zaiter@univ-cotedazur.fr (H.S.-Z.); kevin.gemy@outlook.fr (K.G.); camille.lyko@cea.fr (C.L.); melanie.boudaud@cea.fr (M.B.); morgane.desseux@cea.fr (M.D.); christel.marquette@cea.fr (C.M.); tiphaine.barjat@chu-st-etienne.fr (T.B.); nadia.alfaidy-benharouga@cea.fr (N.A.); 2 Commissariat à l’Energie Atomique (CEA), DSV-IRIG, F-38054 Grenoble, France; 3 University of Grenoble Alpes (UGA), F-38058 Grenoble, France

**Keywords:** prion protein (PrP^C^), cancer, drug resistance, therapeutic target

## Abstract

**Simple Summary:**

Despite its involvement in several human pathophysiological processes, the cellular prion protein (PrP^C^) remains enigmatic. During the last ten years, PrP^C^ has also been reported to be implicated in several human cancers, the molecular mechanisms of which are under investigation. In some tumors, elevated expression of PrP^C^ protein is associated with poor patient prognosis. At the cellular level, high PrP^C^ expression in tumor cells is associated with the acquisition of stemness1-like characteristics, metastatic and invasive process, and resistance to chemotherapy. This review explores PrP^C^’s expression in different types of cancer and addresses its potential as a target for their treatment.

**Abstract:**

The cellular prion protein (PrP^C^) is a glycoprotein anchored to the cell surface by glycosylphosphatidylinositol (GPI). PrP^C^ is expressed both in the brain and in peripheral tissues. Investigations on PrP^C^’s functions revealed its direct involvement in neurodegenerative and prion diseases, as well as in various physiological processes such as anti-oxidative functions, copper homeostasis, trans-membrane signaling, and cell adhesion. Recent findings have revealed the ectopic expression of PrP^C^ in various cancers including gastric, melanoma, breast, colorectal, pancreatic, as well as rare cancers, where PrP^C^ promotes cellular migration and invasion, tumor growth, and metastasis. Through its downstream signaling, PrP^C^ has also been reported to be involved in resistance to chemotherapy and tumor cell apoptosis. This review summarizes the variance of expression of PrP^C^ in different types of cancers and discusses its roles in their development and progression, as well as its use as a potential target to treat such cancers.

## 1. Introduction

### 1.1. Background

**Figure 1:**
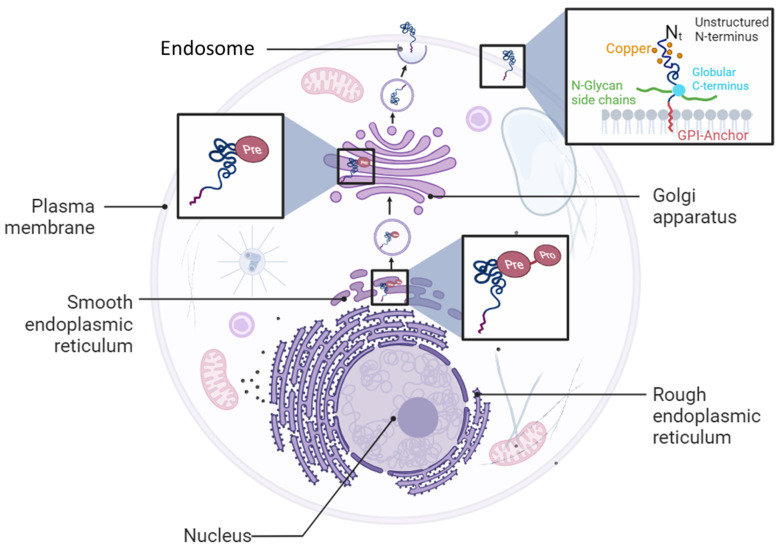
Cellular biosynthetic pathway of PrP^C^ protein. PrP^C^ is synthetized as a pro-pre-protein in the endoplasmic reticulum compartment before trafficking to the Golgi apparatus and plasma membrane where it is anchored as a glycosylphosphatidylinositol (GPI) protein.

Cellular prion protein (PrP^C^) is a glycosylphosphatidylinositol (GPI)-anchored glycoprotein expressed on the cell surface in various organs and tissues [1]. The PrP^C^ protein is encoded by the *PRNP* gene that is localized on Chromosome 20 and 2 in humans and in mice, respectively [1]. PrP^C^ is first synthesized as a pre-pro-protein with a leader peptide at the N-terminal tail, and a GPI anchor signaling peptide (GPI-PSS) at the C-terminal tail. The leader peptide guides the pre-pro-PrP^C^ into the endoplasmic reticulum (ER) where it is cleaved to generate the pro-PrP^C^ (Figure 1). Like other GPI-anchored proteins, pro-PrP^C^ is then translocated from the ER to the Golgi with the help of post-GPI attachment proteins 1 and 5 (PGAP1 and PGAP5) [2,3]. In this compartment, PrP^C^ becomes a mature protein by undergoing further processes such as N-linked glycosylation, GPI-PSS removal, and addition of the pre-assembled GPI anchor [4,5] (Figure 1). PrP^C^ is translocated from the Golgi to the outer leaflet of the plasma membrane where it is inserted via its GPI domain [4,5] (Figure 1).

**Figure 2:**
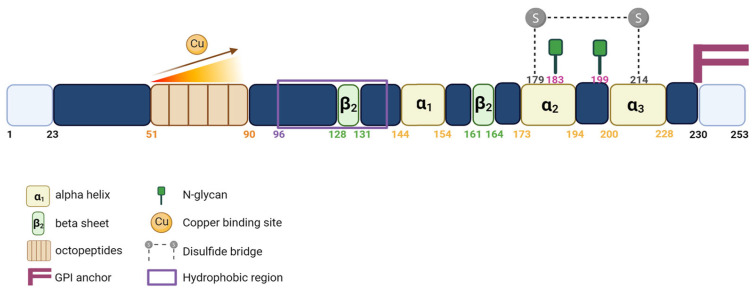
Schematic representation of the PrP^C^ protein. Linear representation of the primary sequence of human PrP^C^ showing important protein domains.

PrP^C^ contains a flexible N-terminal domain (Nt) located between residues 23 and 124. It comprises five repetitive motifs of eight amino acids (PHGGGWGQ) that exhibit a high affinity for copper ions (Cu^2+^). This binding takes place within the HGGGW residues that showed, in vitro, more affinity for Cu^2+^ than for Cu^+^ or to any other metal ion [6] (Figure 1 and Figure 2). PrP^C^ also exhibits a globular C-terminal domain (Ct), anchored to the plasma membrane, of about 100 amino acids, from residues 125 to 228. This domain is composed of three α-helices, corresponding to amino acids 144–154, 173–194, and 200–228, interspersed by two antiparallel β sheets of residues 128–131 and 161–164 (Figure 2). 

The third α helix and the second β sheet are connected by a flexible loop. There are two N-glycosylation sites (residues 183 and 199), which might not be partially or fully glycosylated, resulting in three distinct forms of the PrP^C^: the non-glycosylated (~25 kDa), the mono-glycosylated (~25 to 30 kDa) and the bi-glycosylated forms (~35 kDa), respectively. PrP^C^ is also characterized by the presence of a single disulfide bridge between the two cysteine residues 179 and 214, which allows the link between helix 2 and 3 and serves to stabilize the tertiary structure of the PrP^C^ (Figure 2). Between the Nt and Ct domains (residues 110 to 135), there is a strongly conserved hydrophobic region called the TMD (transmembrane domain). The function of this region is not yet known, but seems to be involved in the conversion of PrP^C^ to a pathogenic form [7]. PrP^C^ exhibits a highly conserved structure in mammals regardless of the degree of the sequence’s homology.

### 1.2. PrP^C^ Expression and Functions

The expression of PrP^C^ begins at embryogenesis [1]. The highest level of PrP^C^ expression was found in the central and peripheral nervous systems [1]. In adults, strong expressions were detected in the brain, spinal cord, neurons, and glial cells [8,9]. PrP^C^ expression was also ubiquitously detected in various cells of the peripheral tissues [10,11,12,13].

The interest in the study of PrP^C^ was mainly related to its incrimination in the pathogenesis of the neurodegenerative disorders known as spongiform encephalopathies (SE) or prion diseases [14,15]. 

The SE was mainly associated with bovines (BES), and commonly referred to as mad cow disease, which refers to an untreatable and inevitably fatal neurodegenerative illness that affects cattle [16]. BES is characterized by the aggregation of an abnormal beta-sheet rich isoform of the PrP^C^ protein called scrapie (PrP^Sc^) [14,15]. In humans, the corresponding form of BES is Creutzfeldt-Jakob’s Disease (CJD), which is also characterized as a brain degenerative disorder [17]. Fatal familial insomnia (FFI) stands as an exceptionally rare prion disease that induces neurodegeneration and primarily manifests through insomnia, making it incredibly challenging to sleep. The predominant instances of this condition are hereditary in nature, resulting from a mutation in the PRNP gene, while sporadic cases make up the remaining occurrences [18]. Scrapie gives rise to a lethal degenerative ailment that targets the nervous systems of goats and sheep. Classified as one of several transmissible spongiform encephalopathies (TSEs), it is believed to stem from prions [19]. Numerous other prion diseases affect both animals and humans, including kuru, Gerstmann–Sträussler–Scheinker syndrome, and chronic wasting disease. These disorders are characterized by prolonged incubation periods, abnormal behavior, and rapid deterioration of brain function. Unfortunately, they always culminate in fatality and currently lack any known cure. 

In addition to its involvement in prion diseases, several studies have attributed plenty of physiological roles to PrP^C^ including anti/pro-apoptosis, metal homeostasis, anti-oxidative damage, cell adhesion and migration, signaling, immune modulation, cell differentiation, and epithelial junctions [20,21,22,23,24,25,26,27]. Yet, the PrP^C^ physiological function is still enigmatic, since no obvious phenotype was observed in PrP^C^ knockout mice [28,29].

In the last decade, PrP^C^ has also been shown to play a significant role in cancer biology. PrP^C^ has been found to be upregulated or ectopically expressed in different types of cancer tissues, such as hepatocellular carcinoma, gastric cancer, melanoma, breast cancer, colorectal cancer, pancreatic ductal adenocarcinoma, prostate cancer, osteosarcoma, and glioblastoma [30,31,32,33,34,35,36,37,38,39]. The increased expression of PrP^C^ appears to play a crucial role in cancer growth, development, differentiation, invasion, migration, metastasis, chemotherapy resistance, and resistance to apoptosis [30,31,32,33,34,35,36,37]. The growing body of evidence linking PrP^C^ to cancer has opened up new avenues for cancer research [40,41]. 

The interaction of PrP^C^ with various proteins and receptors leads to the activation of intracellular signaling pathways that promote tumorigenesis [42,43]. The differential expression of PrP^C^ in various types of cancer, its involvement in protein–protein interactions and its activation of downstream pathways confers to this protein a likely role in cancer (Table 1).

While much progress has been made in the last few years, there is still much to do to fully understand the role of PrP^C^ in cancer and to develop effective therapies targeting this protein. One promising area of research is the development of new compounds that can directly reduce the levels of expression of PrP^C^ in cancer cells [74]. 

These compounds have shown great potential in preclinical studies, and could represent a new class of anti-cancer agents. Additionally, the development of monoclonal antibodies against PrP^C^ and PrP^C^-specific T cells represents exciting new approaches for cancer immunotherapy [75].

## 2. Aims

While much remains to be done, the growing body of evidence suggests that PrP^C^ is a promising target for cancer treatment and that sustained research in this area is warranted. Understanding the mechanisms by which PrP^C^ contributes to cancer progression is of major interest that may help to develop new and more effective therapies targeting this protein. With continued effort and innovation, PrP^C^ could become an important target for cancer treatment in the years to come.

Our review aims to create a summary of the specific roles of PrP^C^ in each type of cancer and discuss the underlying mechanisms that may shed light on potential cancer-targeted therapy involving this protein.

## 3. PrP^C^ in Human Cancers

### 3.1. PrP^C^ and Gastric Cancer 

The expression of PrP^C^ has been reported to be highly elevated in gastric cancer tissue, indicating its potential involvement in the pathogenesis of this disease [31]. Moreover, PrP^C^ has been shown to promote multidrug resistance in gastric cancer cells by inhibiting apoptosis [31]. Due to its ability to bind to certain extracellular matrix and adhesive proteins, PrP^C^ exhibits an adhesive feature, indicating its involvement in cell adhesion [33,37,76]. A comparison of the PrP^C^ expression in primary and metastatic sites was conducted in patients with metastatic and non-metastatic gastric cancer [34]. Although no significant difference in the PrP^C^ expression was observed between the primary and metastatic sites, a higher staining score for PrP^C^ was observed in the metastatic compared to the non-metastatic cancers, indicating a potential correlation between the PrP^C^ expression and gastric cancer aggressiveness. Moreover, the expression of the PrP^C^ protein has been shown to enhance the adhesive, invasive, and metastatic abilities of cancer cells through the activation of the ERK1/2 signaling pathway and transactivation of MMP11, a metalloproteinase responsible for extracellular matrix (ECM) degradation in cancer [34,36]. In vitro invasion assays showed a strong invasiveness profile of two gastric cancer cell lines that constitutively express PrP^C^ (SGC7901 and MKN45), confirming the role of PrP^C^ in the invasion process. Additionally, using a tail vein metastasis model, these cell lines exhibited metastatic capacity to the liver and other organs [34]. To validate the role of PrP^C^ in the development of gastric cancer, its expression was downregulated using siRNA vectors named PrPsi1. The knockdown of PrP^C^ resulted in a decrease in the adhesive and invasive abilities of both SGC7901 and MKN45 cells, and to a reduction in the metastasis process in vivo [34]. Mechanistically, the metastatic potential of PrP^C^-expressing gastric cancer cells is mediated by MMP11 [36,44]. Inhibition of MMP11 using an anti-MMP11 antibody decreased the number of invasive cells in a concentration-dependent manner [34]. Further studies revealed that the NH_2_-terminal region of PrP^C^ was critical for conferring invasive properties to gastric cancer cells, by using the ERK1/2 signaling pathway [34].

Molecular studies using three different PrP^C^ constructs, NH2-terminal deleted (PrPΔN), Octarepeat-copper binding region (PrPΔOR), and C-terminal deleted (PrPΔC), confirmed the critical role of the N-terminal region of the PrP^C^ protein in promoting the invasive properties of gastric cancer cells [34,77]. Therefore, these findings suggest that PrP^C^ plays a significant role in promoting the adhesive, invasive, and metastatic abilities of gastric cancer cells and that targeting PrP^C^ or its downstream effectors may represent a potential therapeutic strategy for gastric cancer.

### 3.2. PrP^C^ and Melanoma

Previous studies have shown that PrP^C^ interacts with Filamin A (FLNA) to promote cancer progression [47]. PrP^C^-silenced FLNA-deficient M2 melanoma cells exhibited decreased M2 cell migration in wound healing assays [21]. This was further reversed by reintroducing PrP^C^ in PRNP-null M2 cells [46]. Despite the fact that PrP^C^ enhances cell migration and alters the cell cytoskeleton organization through FLNA disruption, M2 cells do not express FLNA. Indeed, the effect of PRNP deletion on cell migration was shown to be associated with F-actin protein. The latter, in wild-type M2 cells that are characterized with a higher mobility, shows an expression level which varies according to that of PrP^C^ [46]. These findings demonstrate that PrP^C^ negatively regulates F-actin without binding to FLNA. To determine the pathway through which PrP^C^ affects F-actin, Hsp27 was assessed based on its importance for cell motility and its ability to reduce actin aggregation [78,79]. The levels and phosphorylation of Hsp27 were evaluated in the presence or absence of PrP^C^. There was a significant decrease in phosphorylated Hsp27 at Ser82 when PRNP was deleted, and P-Hsp27 levels were rescued when PRNP was re-expressed in PRNP-null M2 cells [46]. To identify the kinase responsible for this observation, the inhibition of P38MAPK, Akt, PKD, PKA, and PKC was assessed, as these kinases have been reported to act on Hsp27 [80,81,82,83,84]. Only Akt inhibition decreased the P-Hsp27 levels that were also decreased when PRNP was silenced. The Akt expression was rescued when PrP^C^ was re-expressed. The binding between Akt and Hsp27 was confirmed by co-immunoprecipitation and co-purification in the presence of PrP^C^ and was higher in comparison to their binding in PrP^C^-null M2 cells [46]. These findings support the correlation between PrP^C^ and Akt levels, which will disturb the downstream Akt/Hsp27 interaction, inducing the regulation of actin polymerization and cell migration. PrP^C^ interaction with FLNA also promotes FLNA interaction with β1 integrin, contributing to melanomagenesis [45,48]. A7 cells, which express FLNA, exhibited higher spreading and migration ability compared to M2 cells that do not express FLNA [85]. PrP^C^ exists as Pro- PrP^C^ in both A7 and M2 cells, retaining its glycosylphosphatidylinositol anchor peptide signal sequence (GPI-PSS) with an FLNA binding motif. Reducing the PrP^C^ expression in A7 cells altered the distribution of FLNA and the organization of actin, diminishing cell migration. Integrin β1 also binds FLNA as an independent complex from PrP^C^-FLNA, but reducing the PrP^C^ expression caused a decrease in FLNA-Integrin β1 binding. Therefore, in A7 cells, FLNA interacts with Integrin β1, which is enhanced by Pro-PrP^C^, leading to spreading and migration. 

The in situ detection of Pro-PrP^C^ in melanoma and its increased expression in invasive melanoma indicates that PrP^C^ is directly involved in the development of this cancer [45,48].

### 3.3. PrP^C^ and Breast Cancer

The resistance of cancer cells to apoptosis or drug treatment is one of the main features of tumorigenesis. Epigenetic modifications [86], ectopic gene expression [50,87,88], and oncogene overexpression can lead to aberrant expression of anti- or pro-apoptotic proteins. In breast cancer, PrP^C^ has been reported to contribute to cancer resistance to apoptosis and drug treatment. Chemotherapy of TNF-resistant breast carcinoma cells was effective in patients who were PrP^C^-negative. However, PrP^C^ overexpression in estrogen receptor (ER)-negative breast cancer patients was linked to decreased sensitivity to chemotherapy, indicating that PrP^C^ could potentially be used as a predictor of adjuvant chemotherapy benefit in ER-negative patients [50].

Overexpression of PrP^C^ has also been shown to cause resistance to TRAIL (Tumor necrosis factor-Related Apoptosis Inducing Ligand)-induced apoptosis in Adriamycin (MCF7/ADR) [49,51,52,53,54,55]. The elevated expression of PrP^C^ in MCF7/ADR and 2101 cell lines compared to MCF7 cells correlates with the breast carcinoma cells’ resistance to Adriamycin and TRAIL-induced cell death [89]. 

**Figure 3:**
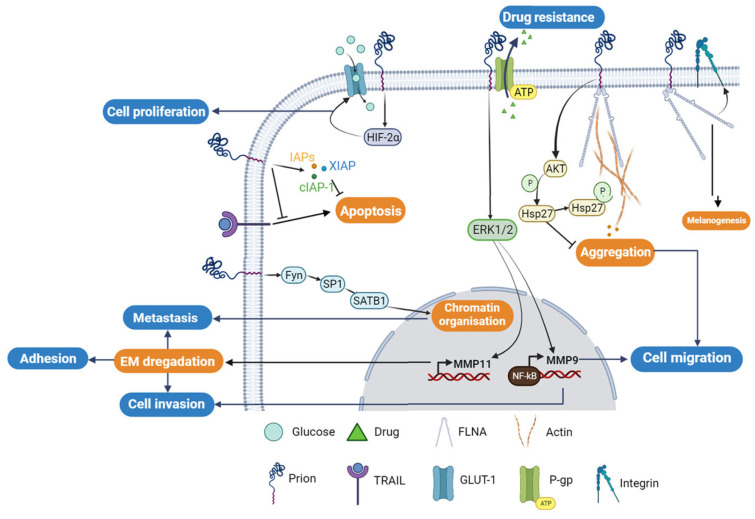
Cellular regulatory pathways involving PrP^C^ protein in cancer. The PrP^C^ protein is involved in different cellular tumorigenesis process, where its activities are regulated directly through protein–protein interactions or/and indirectly using different cellular regulatory pathways. P-gp; P-glycoprotein, GLUT-1; Glucose transporter-1; FLNA; Filamin A.

Nevertheless, the knockdown of PrP^C^ using the siRNA-PrP^C^ strategy in resistant cell lines only restores sensitivity to TRAIL-mediated apoptosis by up to 25% in MCF7/ADR and 60% in 2101 cells. This is achieved through the enhancement of Bid cleavage and caspase-3 processing, concomitantly with Mcl-1 downregulation and activation of pro-apoptotic Bax through the downregulation of Bcl-2 [89]. In addition to its role in acquiring resistance, PrP^C^ has been shown to be a crucial factor for invasion and migration of MCF7 breast cancer cells. PrP^C^ overexpression increases matrix metalloprotease-9 (MMP-9) expression by enhancing the association of NF-κB with the promoter of the MMP-9 gene and ERK signaling, similar to that observed in gastric cancer [57] (Figure 3). Furthermore, PrP^C^ physically associates with P-glycoprotein (P-gp), an ATP-binding cassette (ABC) drug efflux pump, leading to higher invasive capacity and advanced malignancies in MCF7/ADR cells treated with paclitaxel [58] (Figure 3). Indeed, paclitaxel had no effect on the invasion of P-gp (+)/PrP^C^ (−) and P-gp (−)/PrP^C^ (+) cells, confirming that this drug promotes the invasion in multidrug-resistant (MDR) breast cancer cells through a mechanism that involves the interaction of P-gp with PrP^C^ [58] (Figure 3).

### 3.4. PrP^C^ and Colorectal Cancer

Colorectal adenocarcinoma (CRC) cells exhibit high levels of expression of PrP^C^ compared to normal colorectal cells. PrP^C^ plays a crucial role in tumor growth and survival by promoting the Warburg effect, which involves increased reliance on glucose metabolism, in the presence of oxygen. This process ensures rapid proliferation and survival of cancer cells [59,61]. Through the Fyn-HIF-2α pathway, PrP^C^ increases the expression of GLUT-1, the main glucose transporter, thereby enhancing the dependency of CRC cells on the glycolytic pathway for tumor growth (Figure 3). In contrast, the depletion of PrP^C^ suppresses glucose utilization by suppressing GLUT-1 expression, leading to the inhibition of tumor growth both in vitro and in vivo [60]. Cell surface proteomics studies have identified the differential expression of GLUT-1 and PrP^C^ as potential biomarkers of colorectal adenoma to carcinoma progression. Hence, these proteins can serve as potential targets for the emerging molecular imaging modalities [90]. Functional assays have revealed a molecular mechanism that links the levels of PrP^C^ expression to the regulation of CRC metastasis. Ectopic PrP^C^ expression was found to promote the in vitro metastatic potential of CRC cells, while inhibition of PrP^C^ significantly reduced cancer cell motility [63]. The pathway involving PrP^C^-mediated upregulation of SATB1 is a matrix attachment region-binding protein that regulates higher-order chromatin organization and tissue-specific gene expression. This pathway uses a novel PrP^C^-dependent pathway that involves the activation of Fyn-SP1-SATB1 complex protein. The depletion of PrP^C^ abolished the activity of Fyn and SP1, resulting in reduced SATB1 expression [63]. PrP^C^ has also been found to increase the growth of LS-174T colon cancer cells and promote their invasion and migration abilities [56]. Additionally, cancer stem cells expressing CD44^+^/PrP^C+^ exhibited a higher liver metastatic capacity compared to CD44^+^/PrP^C-^ stem cells from CRC, emphasizing the contribution of PrP^C^ to cancer metastasis [62]. Recently, PrP^C^ was shown to interact with c-Met in colorectal cancer cells to regulate cancer stem cell properties [91].

In colorectal and pancreatic ductal adenocarcinoma (PDAC), the overexpression of PrP^C^ has been shown to confer resistance to anti-cancer drugs, including doxorubicin, etoposide, and vincristine sulfate [64,65,66,67]. In LS-174T cells overexpressing PrP^C^, a higher cell viability and less apoptosis were observed compared to non-transfected cells. The PrP^C^ anti-apoptotic effect is thought to be mediated through the upregulation of the three proteins that are involved in the inhibition of apoptotic pathway. These include the inhibitor of apoptosis proteins (IAPs)-survivin, the X-linked inhibitor of apoptosis protein (XIAP), and the cellular inhibitor of apoptosis protein-1 (cIAP-1) [56] (Figure 3). On the other hand, the silencing of PrP^C^ has been shown to enhance the anti-cancer effect of fucoidan in HT29 colon cancer cells [67]. 

Fucoidan (a sulfated polysaccharide with anti-inflammatory and anti-cancer properties) treatment led to reduced PrP^C^ expression, which results in an anti-proliferative and pro-apoptotic effect. When PrP^C^ expression was further downregulated using siRNA, in addition to fucoidan treatment, a further increase in apoptotic cells and a significant reduction in cell migration were observed [67].

At the molecular level, it was proposed that the PrP^C^ involvement in PDAC is mediated upon its interaction with filamin A (FLNA). This interaction affects the cytoskeleton organization and the expression of different signaling proteins, triggering the cellular proliferation and invasiveness, leading to overall tumor growth [64,66] (Figure 3). In addition, the expression of PrP^C^ in PDAC has been associated with a poor prognosis and reduced patient survival. One study found that the risk of death was four times higher (HR = 3.8; 95% CI: 2.2, 6.5) in 108 PDAC cases with PrP^C+^ tumors (median survival 5 months) compared to the 34 cases with PrP^C-^ tumors (median survival 20 months), indicating that PrP^C^ may serve as a potential prognostic biomarker of PDAC [67]. Hence, targeting PrP^C^ could be a potential therapeutic approach to overcoming drug resistance and improve the efficacy of anti-cancer treatment.

## 4. The Potential Diagnostic and Therapeutic Value of PrP^C^ in Different Types of Cancer

PrP^C^ expression has been investigated in various types of cancer, including bladder and prostate cancer, osteosarcoma, and glioblastoma [41,68,69,70,92]. In prostate spheroids, PrP^C^ expression was inversely correlated with the spheroid diameter and related to the intracellular redox state, potentially by contributing to anti-oxidative defense. Moreover, PrP^C^ was found to be overexpressed in 90% of prostate cancer biopsies, although its diagnostic or prognostic value remains unknown [68]. In osteosarcoma, the most common bone malignancy, PrP^C^ was differentially overexpressed and appeared to be associated with tumor development and aggressiveness, as well as a negative regulator of apoptosis [69]. In glioblastoma, a CNS solid tumor, PrP^C^ was highly expressed and found to contribute to tumorigenesis through its interaction with the stress-inducible protein-1 STI1 [70]. 

PrP^C^ expression was directly correlated with the proliferation of glioma stem cells (GSC), and its downregulation reduced GSC stemness, cell growth, clonogenicity, and spherogenicity, as well as the ability to develop tumors in animal models. The results imply that PrP^C^ plays a crucial role in preserving GSC stemness [93,94]. 

Hence, blocking its activity could enhance the sensitivity of cancer cells to chemotherapy [70,95]. Interestingly, PrP^C^ expression was found to increase the sensitivity to doxorubicin in MDA-MB-435 breast cancer cells, unlike colorectal cancer, suggesting a tumor type-specific mechanism [71]. 

Recent studies have also shown that PrP^C^ is expressed in human lung epithelial cells and is involved in anti-oxidative defense and the maintenance of tight junctions in the epithelial barrier [72]. Furthermore, PrP^C^ has been reported to be implicated in the invasiveness and metastasis of lung cancer [73], highlighting its crucial role in both lung physiology and lung tumorigenesis, as observed in other types of cancer.

Given the well-known association between smoking and lung cancer, it would be interesting to investigate the effect of nicotine on the levels of expression of PrP^C^ in lung epithelial cells, and to determine how smoking may affect PrP^C^ expression. Hence, further studies are required to decipher the contribution of PrP^C^ to lung tumorigenesis.

## 5. Targeting PrP^C^ Interactions in Cancer: New Insights and Potential Strategies (Figure 4)

**Figure 4:**
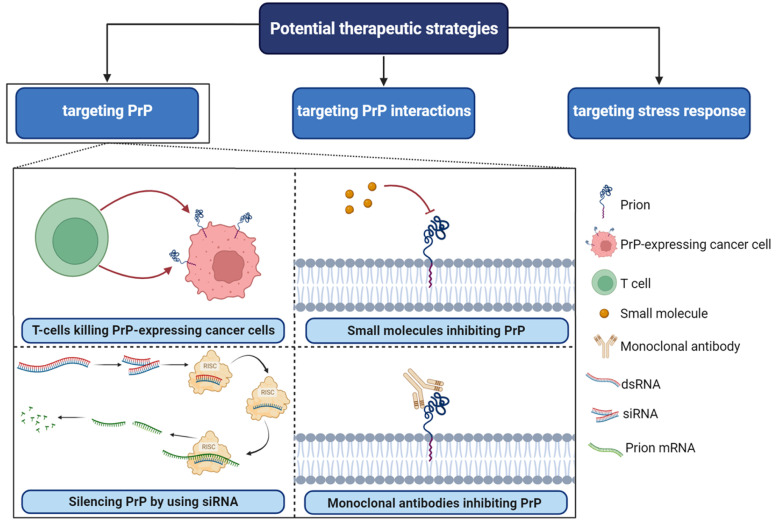
Potential therapeutic strategies in PrP^C^-associated cancer. RISC; RNA-induced silencing complex, SiRNA; small interfering RNA, dsRNA; double-stranded RNA. Several small molecules, including Quinacrine, Chlorpromazine, Amphotericin B, Pentosan polysulfate, and Suramin, have been identified as potential inhibitors of PrP^C^.

PrP^C^ plays a central role as a scaffold protein by forming multiprotein complexes with receptors or extracellular molecules. These interactions may contribute to the activation of downstream signal pathways that control numerous biological functions, including cancer stem cell self-renewal, the central entity of tumor maintenance and dissemination [41,96]. One potential strategy for targeting PrP^C^ in cancer is to disrupt its interactions with other molecules known to be involved in cancer progression [95]. For example, PrP^C^ has been shown to interact with several cell surface receptors, including integrins and laminin receptors. Importantly, these proteins have been reported to play important roles in cancer cell adhesion, migration, and invasion [68]. Inhibiting these interactions could potentially prevent cancer cells from spreading and invading surrounding tissues. In breast cancer, PrP^C^ interaction with P-gp was associated with drug resistance, higher aggressiveness, invasion, and migration. PrP^C^ also interfered in neo-adjuvant chemotherapy response in this cancer [96]. In addition, the PrP^C^-STI1 interaction has been shown to be involved in many tumors, including glioblastoma [96]. In PDAC and melanomas, pro-PrP^C^ has been shown to interact with FLNA to promote tumorigenesis and was associated with worse prognoses. These results strongly suggested that inhibition of these interactions by specific compounds could constitute a promising therapy to treat cancer [97]. Additionally, in some categories of breast cancer that are resistant to conventional treatment, ER stress increased the PrP^C^ expression, contributing to their survival. Therefore, targeting ER stress response and PrP^C^ may provide synergistic effects [98]. PrP^C^ silencing has been shown to sensitize breast cancer cell lines to TRAIL-, Bax, TNF-α, and adjuvant chemotherapy-mediated cell death, which can also be considered as alternative treatments in breast cancer [96] (Figure 3). The direct targeting of PrP^C^ protein, may also be considered as an interesting alternative. Several compounds such as small molecules, peptides, and siRNA, have been identified as reducers of PrP^C^ expression in cancer cells [74]. These compounds have shown promising results in preclinical studies and may represent a new class of anti-cancer agents (Figure 4).

Finally, immunotherapy approaches targeting PrP^C^ have also been explored [99]. For example, monoclonal antibodies against PrP^C^ have been shown to inhibit cancer cell proliferation and migration in vitro and in vivo [99]. Additionally, PrP^C^-specific T cells have been generated and have been shown to recognize and kill PrP^C^-expressing cancer cells. Overall, these studies strongly suggest that the targeting of PrP^C^ interactions with tumor-associated proteins may represent a promising new avenue for cancer therapy. 

While much more research is needed to fully understand the role of PrP^C^ in cancer development and progression and to develop effective therapies targeting this protein, the growing body of evidence suggests that PrP^C^ is a promising target for cancer treatment [100] (Figure 4).

## 6. Conclusions

Despite the lack of solid evidence for the precise physiological role of PrP^C^, its involvement in human diseases, especially cancer, is now well-established. A growing body of evidence linking PrP^C^ to cancer has opened up new avenues for cancer research and treatment.

Through this review, we shed light on the role of PrP^C^ in numerous types of cancers where it is highly expressed. In addition, we believe that PrP^C^ may contribute to tumorigenic processes by regulating tumor cell invasion, migration, and metastasis. PrP^C^ also appears to exhibit anti-apoptotic and drug resistance effects. 

At the therapeutic level, one promising area of research is the development of new drugs that can directly reduce the PrP^C^ expression in cancer cells or target its interactions with other molecules involved in cancer progression. Some of these drugs have shown great potential in preclinical studies, and could represent a new class of anti-cancer agents. Moreover, the development of monoclonal antibodies against PrP^C^ and PrP^C^-specific T cells represents exciting new approaches for cancer immunotherapy.

Future research should focus on a better understanding of the mechanisms by which PrP^C^ contributes to cancer progression, as well as on developing new and more effective therapies targeting this protein. With continued effort and innovation, PrP^C^ could become an important target for cancer treatment in the years to come.

In conclusion, the discovery of the link between PrP^C^ and the etiology of cancer will open up exciting new avenues for cancer research and therapy. While much remains to be done, the growing body of evidence suggests that PrP^C^ is a promising target for cancer treatment. Hence, sustained research in this area is warranted.

## Figures and Tables

**Table 1:** Identified roles of PrP^C^ protein in different types of human cancers.

Cancer Type	Role of PrP^C^	References
Gastric Cancer	Promotion of multidrug resistance	[31]
	Enhancement of adhesive and invasive abilities	[33,34,36,44]
Melanoma	Promotion of cancer migration	[21,45,46,47,48]
	Disruption of Filamin A	[21,45,46,47,48]
Breast Cancer	Resistance to apoptosis and drug treatment	[30,49,50,51,52,53,54,55]
	Promotion of invasion and migration	[56,57,58]
Colorectal Cancer	Promotion of tumor growth via Warburg effect	[59,60,61]
	Enhancement of metastasis	[62,63]
	Confer resistance to anti-cancer drugs	[64,65,66,67]
Prostate Cancer	Potential involvement in tumor development	[68]
Osteosarcoma	Association with tumor development	[69]
Glioblastoma	Enhancement of glioma stem cell proliferation	[70,71]
Lung Cancer	Possible involvement in tumorigenesis	[72,73]

## Abbreviations

ABC	ATP-Binding Cassette
Akt	Protein Kinase B
ATP	Adenosine Triphosphate
BES	Bovine encephalopathies spongiform
CD44	Cluster of Differentiation 44
cIAP-1	Cellular Inhibitor of Apoptosis Protein-1
CJD	Creutzfeldt-Jakob’s Disease
CNS	Central Nervous System
CRC	Colorectal Cancer
CSC	Cancer Stem Cells
Ct	C-terminal domain
Cu2+	Copper ions
dsRNA	Double-Stranded RNA
ECM	Extracellular Matrix
ER	Endoplasmic Reticulum
ERK1/2	Extracellular Signal-Regulated Kinases 1 and 2
FFI	Fatal Familial Insomnia
FLNA	Filamin A
Fyn	Proto-oncogene tyrosine-protein kinase Fyn
GA	Golgi Apparatus
GLUT-1	Glucose Transporter 1
GPI	Glycosylphosphatidylinositol
GSC	Glioma Stem Cells
HIF-2α	Hypoxia-Inducible Factor-2 Alpha
HSP27	Heat Shock Protein 27
HT29	A colorectal carcinoma cell line
IAPs	Inhibitor of Apoptosis Proteins
LS-174T	A colorectal adenocarcinoma cell line
MCF7/ADR	MCF7 Adriamycin-Resistant Cells
MDA-MB-435	A breast cancer cell line
MDR	Multidrug Resistance
MKN45	A gastric cancer cell line
MMP	Matrix Metalloprotease
Nt	N-terminal domain
P38MAPK	p38 Mitogen-Activated Protein Kinase
PDAC	Pancreatic Ductal Adenocarcinoma
PGAP1/PGAP5	Post-GPI Attachment Proteins 1 and 5
P-gp	P-glycoprotein
PKA	Protein Kinase A
PKD	Protein Kinase D
PRNP	Prion Protein Gene
PrPC	Cellular Prion Protein
RISC	RNA-Induced Silencing Complex
SATB1	Special AT-Rich Sequence-Binding Protein 1
SE	spongiform encephalopathies
Ser82	Serine 82
SGC7901	A gastric cancer cell line
siRNA	Small Interfering RNA
Sp1	Specificity Protein 1
TMD	Trans Membrane Domain
TNF-α	Tumor Necrosis Factor Alpha
TRAIL	Tumor Necrosis Factor-Related Apoptosis Inducing Ligand
TSE	Transmissible spongiform encephalopathies
XIAP	X-Linked Inhibitor of Apoptosis
